# Identification, Characterization, and Functional Validation of Drought-responsive MicroRNAs in Subtropical Maize Inbreds

**DOI:** 10.3389/fpls.2017.00941

**Published:** 2017-06-02

**Authors:** Jayaraman Aravind, Sharma Rinku, Banduni Pooja, Mittal Shikha, Shiriga Kaliyugam, Mallana Gowdra Mallikarjuna, Arun Kumar, Atmakuri Ramakrishna Rao, Thirunavukkarasu Nepolean

**Affiliations:** ^1^Division of Genetics, Indian Agricultural Research InstituteNew Delhi, India; ^2^Division of Germplasm Conservation, National Bureau of Plant Genetic ResourcesNew Delhi, India; ^3^Department of Life Sciences, Shiv Nadar UniversityGautam Buddha Nagar, India; ^4^National Phytotron Facility, Indian Agricultural Research InstituteNew Delhi, India; ^5^Centre for Agricultural Bioinformatics, Indian Agricultural Statistics Research InstituteNew Delhi, India

**Keywords:** drought, gene expression, maize, miRNA, mRNA, post-transcriptional changes

## Abstract

MicroRNA-mediated gene regulation plays a crucial role in controlling drought tolerance. In the present investigation, 13 drought-associated miRNA families consisting of 65 members and regulating 42 unique target mRNAs were identified from drought-associated microarray expression data in maize and were subjected to structural and functional characterization. The largest number of members (14) was found in the zma-miR166 and zma-miR395 families, with several targets. However, zma-miR160, zma-miR390, zma-miR393, and zma-miR2275 each showed a single target. Twenty-three major drought-responsive *cis*-regulatory elements were found in the upstream regions of miRNAs. Many drought-related transcription factors, such as GAMYB, HD-Zip III, and NAC, were associated with the target mRNAs. Furthermore, two contrasting subtropical maize genotypes (tolerant: HKI-1532 and sensitive: V-372) were used to understand the miRNA-assisted regulation of target mRNA under drought stress. Approximately 35 and 31% of miRNAs were up-regulated in HKI-1532 and V-372, respectively. The up-regulation of target mRNAs was as high as 14.2% in HKI-1532 but was only 2.38% in V-372. The expression patterns of miRNA-target mRNA pairs were classified into four different types: Type I- up-regulation, Type II- down-regulation, Type III- neutral regulation, and Type IV- opposite regulation. HKI-1532 displayed 46 Type I, 13 Type II, and 23 Type III patterns, whereas V-372 had mostly Type IV interactions (151). A low level of negative regulations of miRNA associated with a higher level of mRNA activity in the tolerant genotype helped to maintain crucial biological functions such as ABA signaling, the auxin response pathway, the light-responsive pathway and endosperm expression under stress conditions, thereby leading to drought tolerance. Our study identified candidate miRNAs and mRNAs operating in important pathways under drought stress conditions, and these candidates will be useful in the development of drought-tolerant maize hybrids.

## Introduction

Drought is one of the prevailing abiotic stresses that affect plant growth, development and grain yield (Ceccarelli and Grando, 1996). In particular, regions with insufficient water are more prone to drought due to uneven changes in weather conditions. Furthermore, shortages of water resources resulting from increasing human needs and growing climatic adversities exaggerate the effects of drought several fold (Rosegrant and Cline, 2003). Development of climate-resilient and drought-tolerant cultivars could help in sustaining food grain production in the present era of climate change. However, drought tolerance is a morphologically, physiologically, and genetically complex trait. Therefore, understanding the underlying molecular basis and regulation of drought tolerance could help in accelerating drought-tolerance breeding programs. Many reports have highlighted and proposed various genetic and molecular mechanisms of drought tolerance in different crops, including model plant systems (Ha et al., 2012; Shikha et al., 2017). Additionally, a functional understanding of stress responsive gene(s) and their regulation patterns can aid in devising new genetic tools (Langridge and Reynolds, 2015).

Post-transcriptional modification of RNAs is one of the major forms of regulation of gene expression and is primarily performed by microRNA (miRNA) molecules (Ding et al., 2009; Zhou et al., 2010). miRNAs belong to a non-coding family of RNAs and are known to play major roles in modulating gene expression to adjust plant metabolism to withstand stresses. The regulatory mechanism of miRNAs involves a change in self-concentration, targeting the mRNA quantity and modifying the mRNA expression *via* miRNA-protein complexes. These, in turn, change the ultimate expression of proteins upon exposure to stress (Ding et al., 2009; Wang B. et al., 2014). In plants, miRNA genes code for long pri-miRNA (primary miRNA) transcripts with imperfect stem-loop secondary structures, which are transcribed by RNA polymerase II (Lee et al., 2004; Xie et al., 2005). These transcripts are processed into ~70-nt pre-miRNAs and subsequently released as miRNA/miRNA duplexes by DCL-1 (dicer-like enzyme 1) in association with a dsRNA binding protein, HYL-1. Such duplexes are methylated by a dsRNA methylase, HEN1, and loaded into AGO1 (Kurihara and Watanabe, 2004; Vazquez et al., 2004; Yu et al., 2005). They are subsequently transported to the cytoplasm with the help of an exportin homolog, the HASTY protein (Bartel, 2004; Park et al., 2005), and cleaved into ~22-nt mature miRNAs. Mature miRNA strands are incorporated into a multiprotein complex, the RNA-induced silencing complex (RISC), where they guide the cleavage of complementary target mRNAs by AGO1, which possesses a PAZ domain and a catalytic PIWI domain (Vaucheret et al., 2004; Baumberger and Baulcombe, 2005).

The involvement of miRNAs in different abiotic stresses has been demonstrated in *Arabidopsis*. Overexpression of miR168, miR171 and miR396 under hypersalinity, mannitol, and cold stress was reported in *Arabidopsis* (Liu et al., 2008). Nevertheless, both the involvement of total miRNAs under drought stress and the systemic expression analysis of their drought-related mechanism are still in progress and necessitate further exploration.

Maize is an important crop in the world, contributing significantly to food and nutritional security. However, the production of maize is most vulnerable to various abiotic stresses, especially drought. To date, functional genomics approaches have revealed large amounts of information on target mRNA control through miRNAs for various traits in maize. The expression of a class III homeodomain-leucine zipper (*HD-Zip III*) protein that functions in asymmetrical leaf development and that of a floral meristem transcription factor, *APETALA2*, responsible for meristem identity were found to be targeted by miR166 and miR172, respectively (Juarez et al., 2004). Similarly, miR156 is reported to target the expression of *tga1* (*Teosinte* glume architecture 1) (Chuck et al., 2007). Studies on differential expression of miRNAs have shed light on the regulatory roles of miRNAs in plant development (Kang et al., 2012; Liu et al., 2014) and stress responses in maize (Zhang et al., 2008; Ding et al., 2009; Wei et al., 2009; Zhai et al., 2013; Wu et al., 2014). Such investigations deciphering the regulatory control between miRNAs and target mRNAs will pave the way for a better understanding of the molecular mechanisms underlying drought stress responses. Differentially expressed genes (DEGs) have been identified in drought or low-moisture stress microarray studies (Yu, 2003; Yue et al., 2008; Hayano-Kanashiro et al., 2009; Li et al., 2009; Marino et al., 2009; Luo et al., 2010; Zheng et al., 2010; Lu et al., 2011; Hansen et al., 2013; Regulski et al., 2013). Such DEGs were found to be associated with drought-related miRNA targets.

Maize germplasm shows great genetic variability involving temperate, tropical, and subtropical groups, as well as dent, flint, semi flint and waxy types within germplasm groups. Efforts have been taken to characterize the drought-responsive miRNAs and target mRNAs in temperate maize germplasm (Li et al., 2013; Wang Y. G. et al., 2014). There have been no reports available on comprehensive characterization of drought-responsive miRNAs and target-mRNAs using subtropical maize germplasm. Therefore, in the present study, we identified putative regulatory miRNAs targeting drought-related mRNAs based on gene expression data from 12 drought/low-moisture stress experiments. The expression patterns of miRNA-target mRNA pairs were validated in the root and shoot tissues of two contrasting subtropical maize inbreds under drought stress. The functional annotation and the role of drought-related miRNA-target mRNA pairs were analyzed. Our study identified the differential interactions of miRNA-target mRNA pairs during drought stress in the tolerant genotype and explained their functional roles in drought tolerance.

## Materials and methods

### Plant growth and experimentation

**Figure 1:**
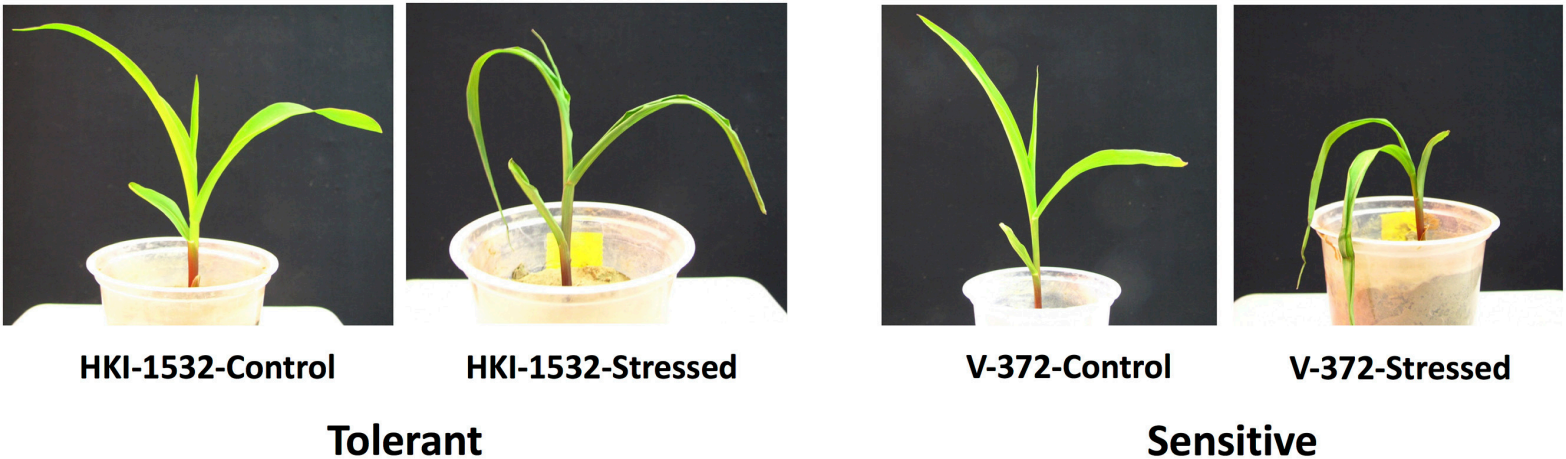
Phenotypic response of tolerant (HKI-1532) and sensitive (V-372) genotypes under drought stress.

Seeds of two contrasting subtropical maize inbreds, HKI-1532 (drought-tolerant) and V-372 (drought-susceptible), were grown in the National Phytotron Facility, Indian Agricultural Research Institute, New Delhi. Potting was done in triplicate with sandy loam soil. The plants were maintained under controlled greenhouse conditions of 28/22°C (day/night) at a light intensity of 600 μmol m^−2^s^−1^ (16 h day/8 h night) with 50–55% relative humidity. Regular irrigation was provided for 15 days to the first set of plants (stress) after sowing and suspended for the next 5 days to induce severe drought stress (Kakumanu et al., 2012; Min et al., 2016; Nepolean et al., 2017). The second set of plants (control) was watered throughout the experiment. On the 21st day after sowing, leaf samples were collected for expression assays (Figure 1).

### Prediction of miRNA-target mRNA interactions

The entire set of maize mature miRNA sequences available (321) was downloaded from miRbase v19 (Griffiths-Jones et al., 2008; Kozomara and Griffiths-Jones, 2011). Four popular miRNA target prediction tools—RNAHybrid (Rehmsmeier et al., 2004), TargetFinder (Allen et al., 2005; http://carringtonlab.org/resources/targetfinder), TAPIR (FASTA search engine) (Bonnet et al., 2010), and psRNATarget (Dai and Zhao, 2011)—were used to identify targets for the miRNAs from among the 76,617 *Zea mays* B73 RegGen v2 mRNAs downloaded from NCBI (*National Center for Biotechnology Information*) Genome database identified by the GNOMON v2 gene prediction tool (Souvorov et al., 2010; ftp://ftp.ncbi.nlm.nih.gov/genomes/PLANTS/Zea_mays/Gnomon_v2/). Consensus predictions of miRNA-target mRNA interactions detected among the four tools were used for further analysis.

### Identification of putative drought-related miRNAs

DEGs identified in 12 microarray-based gene expression studies in maize under drought/low moisture stress and/or recovery irrigation conditions (Table S1) were selected for comparison with the target mRNAs identified in the consensus predictions of miRNA-target mRNA interactions. For the experiments where DEG lists were not available, the raw data from NCBI GEO (*Gene Expression Omnibus*) (Barrett et al., 2005) were reanalyzed to generate a list. The regulatory miRNAs of the DEG sequences that were also predicted to be miRNA targets were identified as putative drought-related miRNAs. For both the mature miRNA sequences and the target mRNA sequences with the respective target sites masked, multiple sequence alignment was done using the Clustal Omega program (Sievers et al., 2011). A distance matrix was constructed based on pairwise distances using an identity matrix with the “dist.alignment” function of the R package seqinR (Charif et al., 2008) and used to performing hierarchical clustering by complete linkage. The clusterings of miRNAs and their respective targets were compared visually by plotting a tanglegram using “*cophyloplot”* function of the R package ape (Paradis et al., 2004). Furthermore, the functional annotation of the corresponding target mRNAs was carried out by Blast2GO (Conesa et al., 2005).

### Analysis of *cis*-regulatory elements (CREs) in miRNA promoters in maize

DNA sequences upstream of the starts of the precursor miRNA structures were identified and extracted using NCBI tools (https://www.ncbi.nlm.nih.gov). The transcription start sites (TSS) were predicted from the upstream regions of pri-miRNA sequences using softberry TSSP (Shahmuradov et al., 2005; http://www.softberry.com/berry.phtml?topic = tss). The promoter sequence, i.e., the 2-kb region upstream of the TSS, was retrieved using Perl Script. For families having >1 promoter, the sequence between the two promoters was considered for the identification of CREs using the PlantCARE (http://bioinformatics.psb.ugent.be/webtools/plantcare/html/) database.

### RNA isolation and qRT-PCR assay

Total RNA was isolated from seedling leaf tissue using the Ultra Clean Plant RNA isolation kit (MO BIO Laboratories, USA). Primers specific to the target mRNA sequences were designed using BatchPrimer3 (You et al., 2008) and optimized to avoid primary and amplicon secondary structures using Beacon Designer^TM^ Free Edition (http://www.premierbiosoft.com/qpcr/) and IDT OligoAnalyzer (http://eu.idtdna.com/analyzer/Applications/OligoAnalyzer/), respectively (Table S2). Sequence specificity of the primers was assured by comparing their sequences with maize gene sequences using NCBI nucleotide BLAST (http://blast.ncbi.nlm.nih.gov/Blast.cgi). qRT-PCR for mature miRNAs was performed using a modified version of stem-loop qRT-PCR (Chen et al., 2005). The stem-loop primers, miRNA forward primers and universal reverse primers were designed according to Kramer (2011) (Table S3). The primer for the reference transcript U6snRNA was designed as previously described (Turner et al., 2013).

For target mRNAs, the first-strand cDNA synthesis was carried out with the RNA ProtoScript First Strand cDNA Synthesis Kit (New England Biolabs, Ipswich, Massachusetts) using 50 ng total RNA and 1 μM Oligo d(*T*)23 VN primer. For miRNAs, reverse transcription reactions were carried out with 50 ng total RNA and 1 μM stem-loop RT primer. The reactions were incubated in a thermocycler for 30 min at 16°C, 30 min at 42°C and 5 min at 85°C and then held at 4°C.

For both mature miRNAs and their mRNA targets, real-time quantitative PCR with SYBR Green I was performed on an Agilent Technologies Mx3005P QPCR (Agilent Technologies, Santa Clara, California, United States) instrument. Briefly, each 25-μl PCR mixture contained approximately 100 ng cDNA, 9 ml Hotstart IT SYBR Green qPCR Master Mix (Affymetrix), ROX (Affymetrix) and 250 nM of each primer. The reactions were mixed gently and incubated at 94°C for 2 min (pre-heating), followed by 40 cycles of 94°C for 15 s (denaturation), 60°C for 30 s (primer annealing), and 72°C for 30 s (primer extension). Analysis was conducted on three biological replicates and two technical replicates for each treatment and genotype combination. U6 snRNA and 18S rRNA were used as internal controls for miRNA and mRNA, respectively. The ΔΔCt method was used to determine the differences in expression levels among samples (Livak and Schmittgen, 2001).

## Results

### Identification of putative drought-related miRNAs and target mRNAs

**Figure 2:**
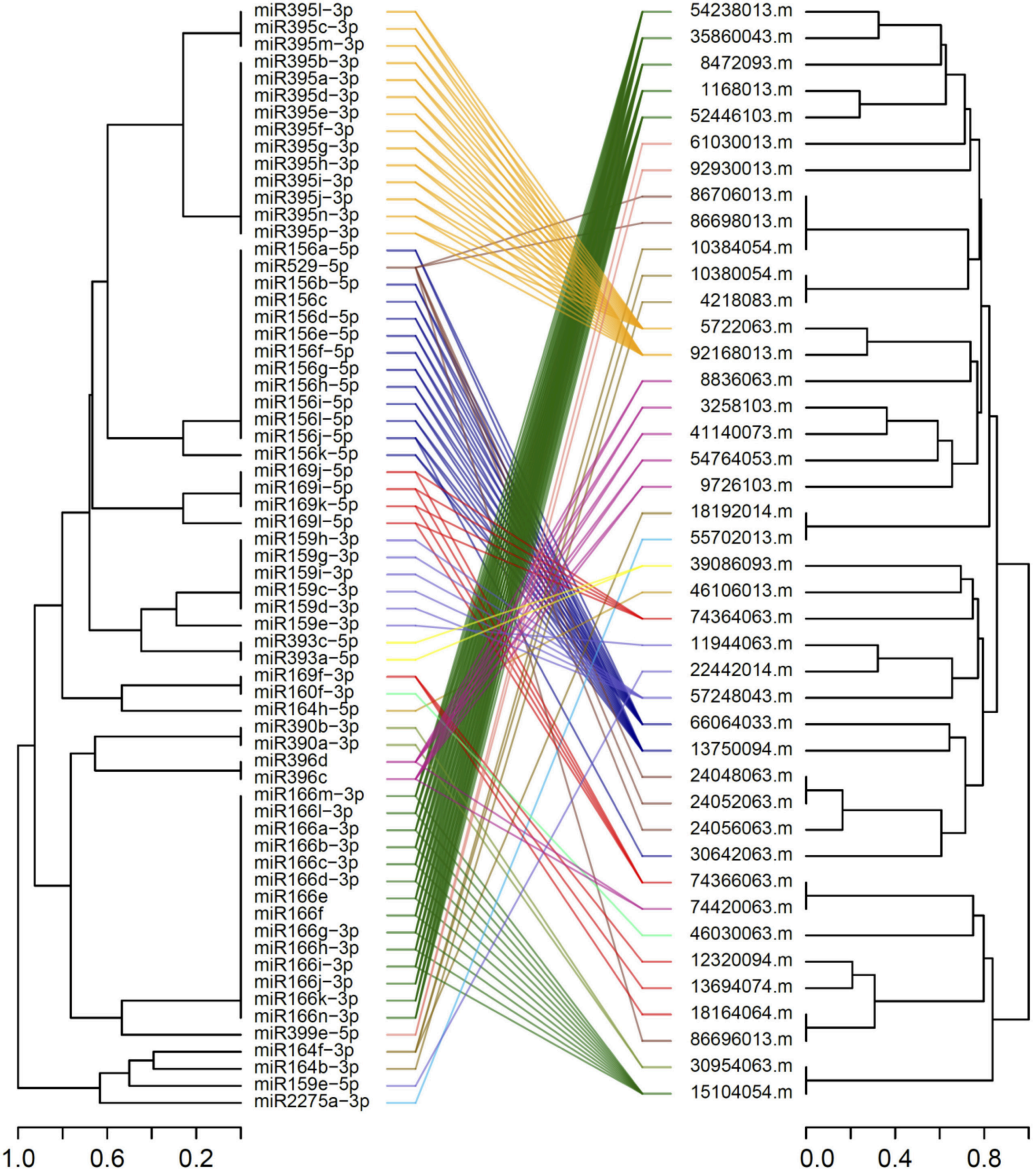
Tanglegram of predicted mature miRNA and target mRNA sequences (with the target site masked) related to drought/low-moisture stress. Lines colored according to the miRNA family, connect the miRNA sequences to their respective target mRNAs.

**Figure 3:**
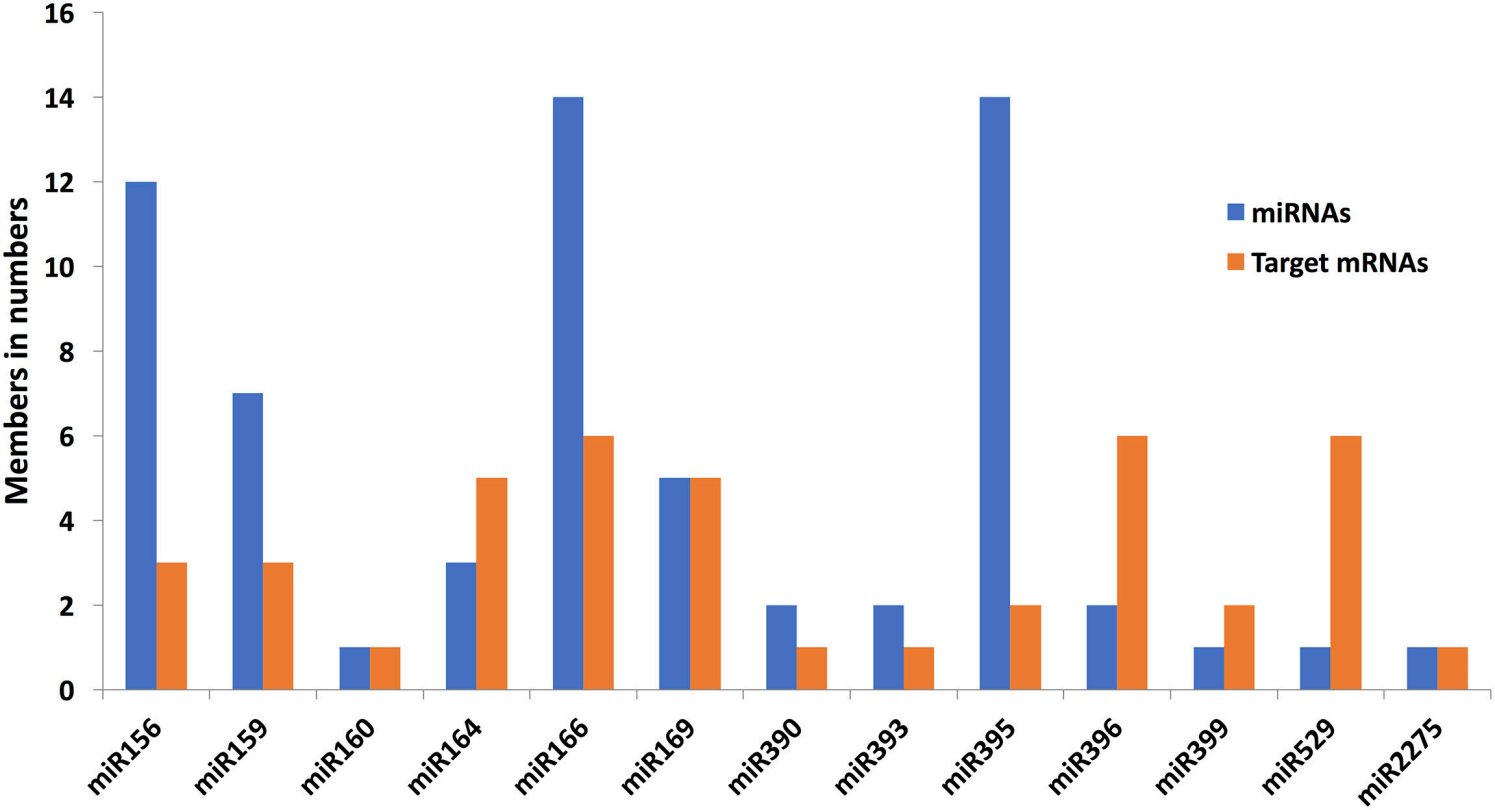
Distribution of 13-drought related miRNA families members and respective target mRNAs. Blue bar represents the members count in each miRNA family and orange bar represents the total number of target mRNAs in each miRNA family.

Among the DEGs identified in 12 microarray-based gene expression studies under drought/low moisture stress and/or recovery irrigation conditions, 42 differentially expressed mRNA sequences were predicted to be putatively regulated by 65 miRNAs belonging to 13 families in 183 miRNA-target mRNA interactions (Table 1). These were considered for further expression analysis. The predicted interactions involved the same miRNAs targeting multiple mRNAs, as well as the same mRNA being targeted by multiple miRNAs. For most of the interactions, miRNAs with similar sequences targeted mRNA sequences that clustered together (Figure 2). The miRNA families with the largest numbers of members included zma-miR166, zma-miR395 and zma-miR156, with 14, 14, and 12 members, respectively. On the other hand, some miRNA families, such as zma-miR160, zma-miR399, zma-miR529, and zma-miR2275, had only one member each (Figure 3). The target distributions of miRNA families themselves were investigated, which showed that zma-miR166, zma-miR396, zma-miR529, zma-miR164, and zma-miR169 had the most targets, with 6, 6, 5, and 5 target mRNAs, respectively, while zma-miR160, zma-miR390, zma-miR393, and zma-miR2275 had the fewest target mRNAs, with a single target each (Figure 3).

**Table 1:** List of 13 drought-related miRNA families and their respective targets with annotations.

**Family specific target mRNAs**			
**miRNA family**	**miRNA**	**Target mRNAs**	**Annotations**
miR156	zma-miR156-a,c,d,e,f,g,h,I,j,k,l	gnl|GNOMON|13750094.m	tpa: squamosa promoter-binding (sbp domain) transcription factor family protein
	zma-miR156-a,c,d,e,f,g,h,I,j,k,l	gnl|GNOMON|66064033.m	Squamosa promoter-binding-like protein 13-like
miR159	zma-miR159e-3p	gnl|GNOMON|11944063.m	Transcription factor gamyb
	zma-miR159e-5p	gnl|GNOMON|22442014.m	Transcription factor gamyb
	zma-miR159-c,d,g,h,i	gnl|GNOMON|57248043.m	Transcription factor
miR160	zma-miR160-f	gnl|GNOMON|46030063.m	gdsl esterase lipase at5g45910-like
miR164	zma-miR164-f	gnl|GNOMON|10380054.m	Hypothetical protein
	zma-miR164-f	gnl|GNOMON|10384054.m	Hypothetical protein
	zma-miR164-f	gnl|GNOMON|18192014.m	Hypothetical protein
	zma-miR164-b	gnl|GNOMON|4218083.m	psbp domain-containing protein
			Chloroplastic-like
	zma-miR164-h	gnl|GNOMON|46106013.m	Wound responsive protein
miR166	zma-miR166-a,b,c,d,e,f,g,h,I,j,k,l	gnl|GNOMON|1168013.m	Rolled expressed
	zma-miR166-a,b,c,d,e,f,g,h,I,j,l	gnl|GNOMON|15104054.m	Partial
	zma-miR166-a,b,c,d,e,f,g,h,I,j,k,m,n,l	gnl|GNOMON|35860043.m	Tpa: homeobox lipid-binding domain family protein
	zma-miR166-a,b,c,d,e,f,g,h,I,j,k,m,n,l	gnl|GNOMON|52446103.m	Rolled leaf1
	zma-miR166-a,b,c,d,e,f,g,h,I,j,k,m,n,l	gnl|GNOMON|54238013.m	Tpa: homeobox lipid-binding domain family protein
	zma-miR166-a,b,c,d,e,f,g,h,I,j,k,m,n,l	gnl|GNOMON|8472093.m	Homeobox-leucine zipper protein athb-15-like
miR169	zma-miR169-I,j,k,l	gnl|GNOMON|74364063.m	Nuclear transcription factor y subunit a-3
	zma-miR169-I,j,k,l	gnl|GNOMON|74366063.m	Nuclear transcription factor y subunit a-3
miR390	zma-miR390-a,b	gnl|GNOMON|30954063.m	Activator of 90 kda heat shock protein atpase
miR393	zma-miR393-a,c	gnl|GNOMON|39086093.m	Protein transport inhibitor response 1-like
	zma-miR393-a,c	gnl|GNOMON|5722063.m	Atpsulfurylase
	zma-miR393-a,c	gnl|GNOMON|92168013.m	Atpsulfurylase
miR395	zma-miR395-b,d,e,f,g,h,I,j,m,n,p,l	gnl|GNOMON|5722063.m	Atpsulfurylase
		gnl|GNOMON|92168013.m	Atpsulfurylase
miR396	zma-miR396-c,d	gnl|GNOMON|3258103.m	Growth-regulating factor 1
	zma-miR396-c,d	gnl|GNOMON|41140073.m	Growth-regulating factor 1
	zma-miR396-c,d	gnl|GNOMON|54764053.m	Growth-regulating factor 1-like
	zma-miR396-c,d	gnl|GNOMON|74420063.m	Growth-regulating factor 9
	zma-miR396-c,d	gnl|GNOMON|8836063.m	Growth-regulating factor 8
	zma-miR396-c,d	gnl|GNOMON|9726103.m	Growth-regulating factor
miR399	zma-miR399-e	gnl|GNOMON|61030013.m	60s ribosomal protein l7a-like
	zma-miR399-e	gnl|GNOMON|92930013.m	Heavy metal-associated domain containing expressed
miR529	zma-miR529	gnl|GNOMON|24048063.m	Squamosa promoter-binding (sbp domain) transcription factor family protein isoform 1
	zma-miR529	gnl|GNOMON|24052063.m	Squamosa promoter-binding (sbp domain) transcription factor family protein isoform 1
	zma-miR529	gnl|GNOMON|24056063.m	Squamosa promoter-binding (sbp domain) transcription factor family protein isoform 1
	zma-miR529	gnl|GNOMON|86696013.m	p8mtcp1
	zma-miR529	gnl|GNOMON|86698013.m	p8mtcp1
	zma-miR529	gnl|GNOMON|86706013.m	p8mtcp1
miR2275	zma-miR2275-a	gnl|GNOMON|55702013.m	Mitochondrial protein

### Prediction of miRNA-target mRNA interactions

**Figure 4:**
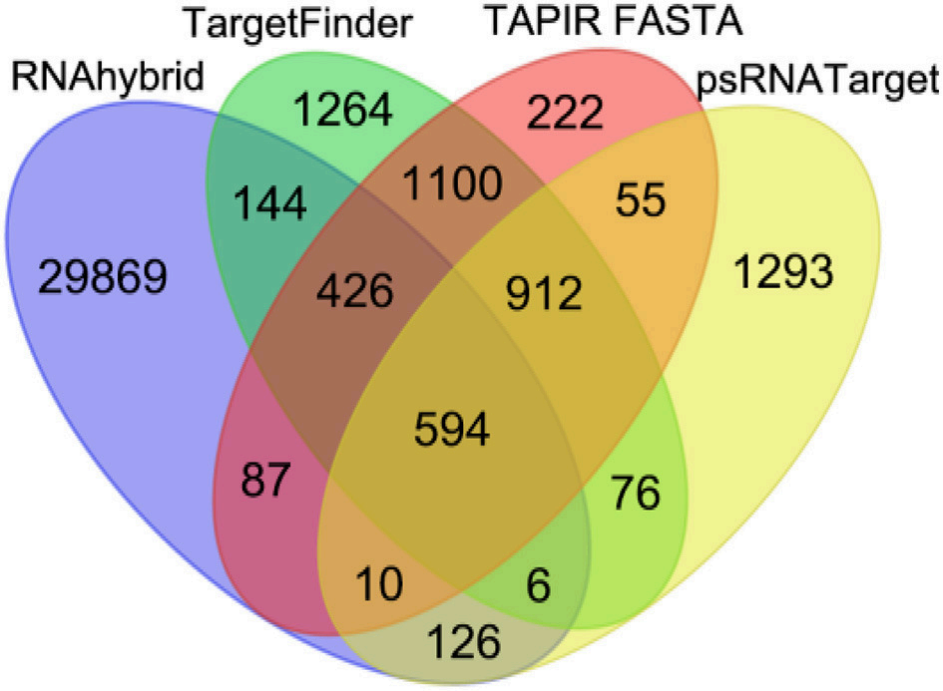
Venn diagram illustrating the number of common and differently identified miRNA-target mRNA interactions through four different source tools (RNAhybrid, TargetFinder, TAPIR FASTA, and psRNATarget). The color of each leaf represents the total and overlapped number of miRNA-target mRNA interactions obtained from each source tool.

The four miRNA target prediction tools detected different but overlapping sets of miRNA-target mRNA pairs from among the 321 miRNAs and 76617 mRNAs of maize. Among these dissimilar sets, 594 consensus predictions of miRNA-target mRNA interactions could be identified. Compared to single prediction tool-based identification of miRNA-target mRNA interactions, we employed four prediction tools to choose the robust miRNA-target mRNA interactions. The total numbers of interactions supported by RNAHybrid, TargetFinder, TAPIR FASTA and psRNATarget were 31,262, 4,522, 3,406, and 3,072, respectively, including both unique and commonly identified interactions from each tool. The numbers of unique interactions identified by RNAHybrid, TargetFinder, TAPIR FASTA and psRNATarget were 29869, 1264, 222, and 1293, respectively (Figure 4). The consensus predictions of miRNA-target mRNAs interactions involved 156 miRNAs belonging to 25 families, and 150 unique target mRNAs.

### GO enrichment analysis of miRNA target genes

**Figure 5:**
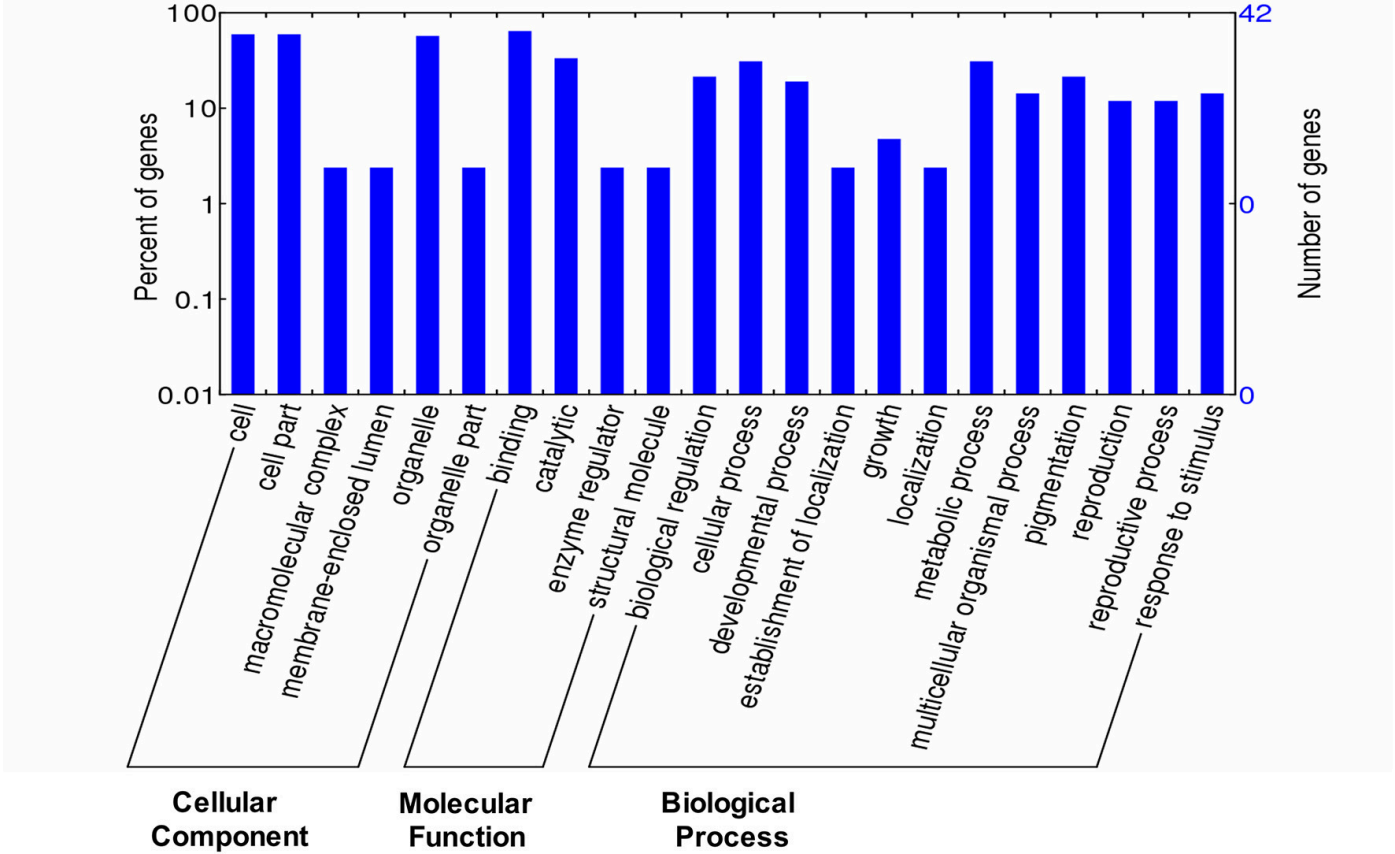
Overview of GO annotations of target mRNAs. The data represents the three major functional categories- biological, molecular, and cellular functions.

To gain insights into the functional roles of miRNAs, annotation of their predicted target mRNAs was carried out. GO terms were identified under three different functional categories: biological (57.14%), cellular (23.80%) and molecular (30.95%). In the biological process category, major functions included anatomical structure morphogenesis, multicellular organismal development and post-embryonic development. In the molecular process category, major functions included DNA binding, hydrolase activity and nucleotide binding (Figure 5). Furthermore, most of the target genes were confined to the nucleus.

### Analysis of *cis-*regulatory elements

The promoter regions of 65 drought-related miRNAs were examined to detect the occurrence of conserved *cis*-regulatory elements. A total of 61 conserved CRE motifs were identified from the miRNA promoters. Among these, 41 were major *cis*-regulatory elements that were observed in >10 members, including the drought-related *cis*-elements ABRE (ABA-responsive element) and MBS (MYB-binding site) (Table 2). In miR169n of rice, the ABRE *cis*-acting element resided in the promoter region, implying an ABA-mediated response to stress (Zhao et al., 2007). An ABRE *cis*-element was also found in sorghum ABA-responsive genes (Buchanan et al., 2005). Similarly, in *Arabidopsis*, MBS was found in the upstream regions of all the miRNA genes in the rice shoot apexes. Of the 65 miRNA members, miR164f showed the highest number of CREs (41), whereas miR156d had the lowest number (8).

**Table 2:** Putative *cis*-regulatory elements identified in the upstream region of drought responsive miRNA in maize.

***cis*-elements**	**Frequency**	**Sequence**	**Function**
TATCCAT/C-motif	10	TATCCAT	*cis*-acting regulatory element; associated with G-box like motif; involved in sugar repression responsiveness
GA-motif	11	AAGGAAGA	Part of a light responsive element
LTR	12	CCGAAA	*cis*-acting element involved in low-temperature responsiveness
CCGTCC-box	12	CCGTCC	*cis*-acting regulatory element related to meristem specific activation
MNF1	12	GTGCCCTT	Light responsive element
ATCT-motif	12	AATCTAATCC	part of a conserved DNA module involved in light Responsiveness
GC-motif	14	CCCCCG	Enhancer-like element involved in anoxic specific inducibility
AE-box	14	AGAAACAT	Expression and repressed
GARE-motif	14	AAACAGA	Gibberellin-responsive element
TCT-motif	14	TCTTAC	Part of a light responsive element
RY-element	15	CATGCATG	*cis*-acting regulatory element involved in seed-specific regulation
5UTR Py-rich stretch	16	TTTCTTCTCT	*cis*-acting element conferring high transcription levels
TCA-element	16	CAGAAAAGGA	*cis*-acting element involved in salicylic acid responsiveness
ACE	17	GACACGTATG	*cis*-acting element involved in light responsiveness
Box I	17	TTTCAAA	Light responsive element
HSE	18	AAAAAATTTC	*cis*-acting element involved in heat stress responsiveness
TGA-element	20	AACGAC	Auxin-responsive element
Box-W1	20	TTGACC	Fungal elicitor responsive element
W box	20	TTGACC	Elicitation; wounding and pathogen responsiveness. Binds WRKY type transcription factors
CCAAT-box	21	CAACGG	MYBHv1 binding site
CATT-motif	21	GCATTC	Part of a light responsive element
O2-site	22	GATGACATGG	*cis*-acting regulatory element involved in zein metabolism regulation
GCN4_motif	22	TGAGTCA	*cis*-regulatory element involved in endosperm expression
Box 4	22	ATTAAT	Part of a conserved DNA module involved in light responsiveness
CAT-box	24	GCCACT	*cis*-acting regulatory element related to meristem expression
GT1-motif	24	GGTTAA	Light responsive element
I-box	24	GATATGG	Part of a light responsive element
AAGAA-motif	26	GAAAGAA	–
TC-rich repeats	28	ATTTTCTTCA	*cis*-acting element involved in defense and stress responsiveness
GAG-motif	30	GAGAGAT	Part of a light responsive element
ABRE	31	GCAACGTGTC	*cis*-acting element involved in the abscisic acid responsiveness
circadian	31	CAANNNNATC	*cis*-acting regulatory element involved in circadian control
ARE	35	TGGTTT	*cis*-acting regulatory element essential for the anaerobic induction
CGTCA-motif	36	CGTCA	*cis*-acting regulatory element involved in the MeJA-responsiveness
TGACG-motif	36	TGACG	*cis*-acting regulatory element involved in the MeJA-responsiveness
Sp1	37	CC(G/A)CCC	Light responsive element
MBS	37	CAACTG	MYB binding site involved in drought-inducibility
G-Box	42	CACGTT	*cis*-acting regulatory element involved in light responsiveness
Skn-1_motif	45	GTCAT	*cis*-acting regulatory element required for endosperm expression
TATA-box	52	ATATAAT	Core promoter element around -30 of transcription start
CAAT-box	53	CCAAT	Common *cis*-acting element in promoter and enhancer regions

### Expression patterns of miRNAs and their target mRNA(s)

**Figure 6:**
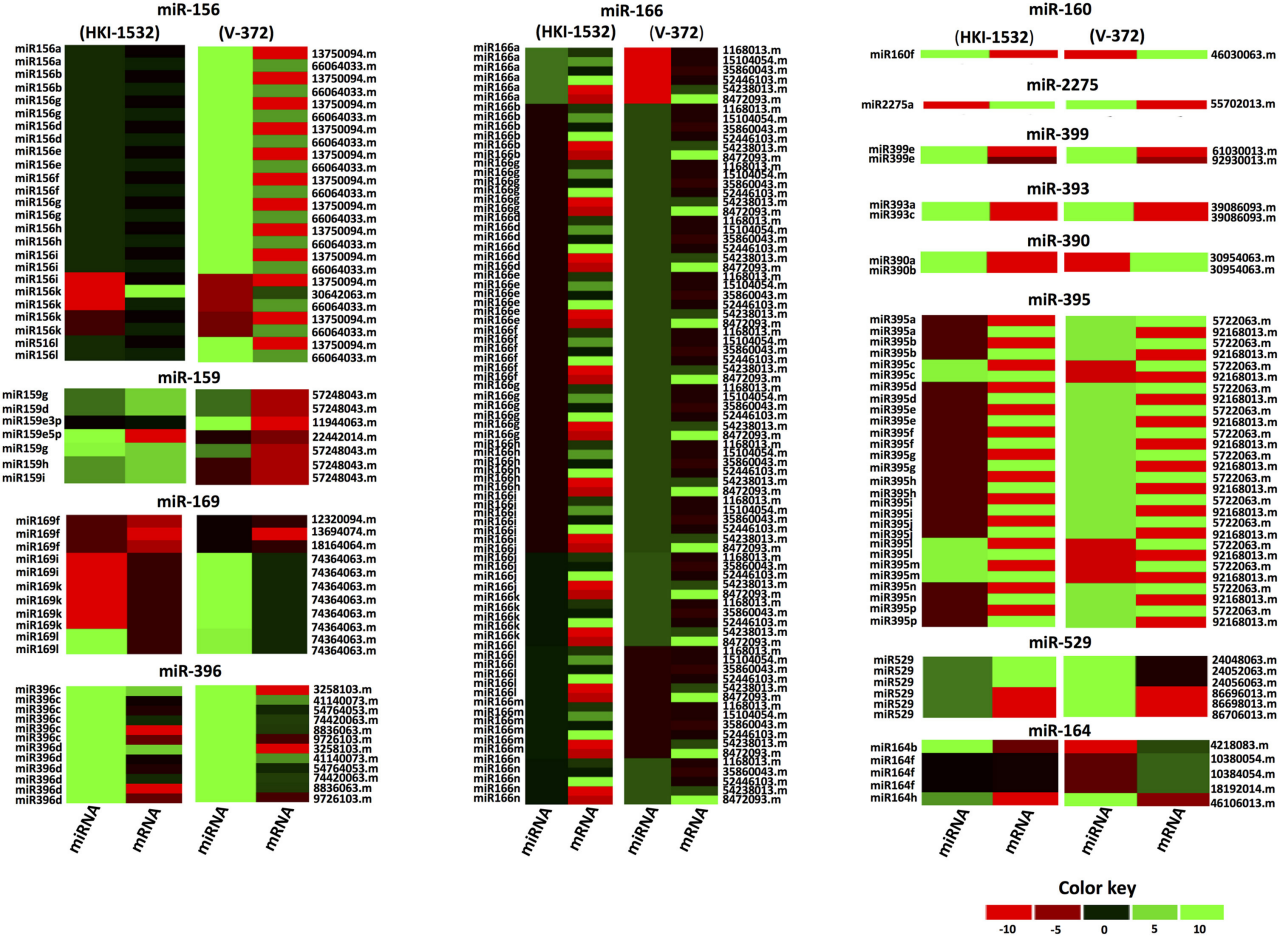
Heat map for altered expression of miRNAs and their respective targets within each family under drought stress. Different color codings have been assigned for specific expression pattern and comparative expression is made in maize genotypes (Left: HKI-1532 and Right: V-372).

To analyze the responses of contrasting genotypes to drought, transcriptional profiling of the identified putative drought-responsive miRNAs and their target-mRNAs was done at the seedling stage, revealing their interactions with each other. The significantly up-regulated miRNAs common to both genotypes (HKI-1532 and V-372) included zma-miR164h, zma-miR169l, zma-miR396c, zma-miR396d, and zma-miR399e. In tolerant genotype HKI-1532, 16 miRNAs belonging to the zma-miR159, zma-miR160, zma-miR164, zma-miR166, zma-miR169, zma-miR390, zma-miR395, zma-miR396, and zma-miR399 families were significantly up-regulated. However, zma-miR156 and zma-miR159 were significantly down-regulated. Conversely, in V-372, members of 7 families—zma-miR164, zma-miR169, zma-miR393, zma-miR396, zma-miR399, zma-miR529, and zma-miR2275—were significantly up-regulated; and zma-miR156, zma-miR159, zma-miR166 and zma-miR395 families were significantly down-regulated. These results indicate disparate patterns of regulation in the tolerant and susceptible genotypes (Figure 6).

Furthermore, expression analysis of target mRNAs revealed six up-regulated, 18 down-regulated and 18 neutrally expressed mRNAs in HKI-1532. V-372, however, had one up-regulated, 10 down-regulated and 26 neutrally expressed mRNAs. The miRNA-target mRNA interactions were classified into four different groups: Type I, with miRNA-mRNA up-regulations; Type II, with miRNA-mRNA down-regulations; Type III, with neutral miRNA-mRNA expressions; and Type IV, with opposite interactions. The tolerant genotype, HKI-1532, had 46 Type I, 13 Type II, 23 Type III, and 101 Type IV combinations. The susceptible genotype, V-372, had eight Type I, four Type II, 20 Type III, and 151 Type IV combinations (Figure 6).

## Discussion

Several investigations have revealed the role of miRNAs in modulating gene expression under abiotic stresses—cold (Lv et al., 2010), salt (Ding et al., 2009), aluminum tolerance (Kong et al., 2014), waterlogging (Zhai et al., 2013), and drought (Li et al., 2013; Wang Y. G. et al., 2014). Among the several abiotic stresses, drought is the most prominent stress in the sub-tropical maize production systems. A number of plant parameters, such as growth, yield, membrane integrity, pigment composition, osmotic relations and photosynthesis, are commonly affected by drought stress (Praba et al., 2009). The change in miRNA expression pattern under drought is an indication of stress responses in plants. Such findings provide an insight into drought tolerance mechanisms and can potentially aid in designing drought-tolerant cultivars (Zhao et al., 2007; Chen et al., 2012). The role of miRNAs in drought tolerance has been previously explored in maize using temperate germplasm. Wang Y. G. et al. (2014) identified 301 drought-responsive miRNAs in temperate maize germplasm and Li et al. (2013) detected differentially expressed 68 miRNAs falling under 29 miRNA families in a maize inbred R09. In the present investigation, 42 differentially expressed mRNA sequences were predicted to be putatively regulated by 65 miRNAs belonging to 13 families. Furthermore, a combination of 183 miRNA-target mRNA interactions were identified and validated in a contrasting pair of subtropical maize germplasm. Four prediction tools were employed to reveal high-confidence predictions of miRNA-target mRNA interactions due to enhanced accuracy and site recognition efficacy as compared to previous single tool-based prediction (Ding et al., 2012; Li et al., 2013; Wang Y. G. et al., 2014). All 13 of the miRNA families were found to be conserved across taxonomic groups related to drought, ABA or oxidative stress response (Kim et al., 2003; Ding et al., 2013). These results suggested that specific microarray expression profiling data could be effectively used for identifying regulatory miRNAs.

miRNA genes originated from the preexisting genes through duplication events (Nozawa et al., 2012). miRNAs that are evolutionarily conserved are usually encoded by miRNA gene families. This, coupled with the strong similarity requirements of plant miRNA - target site interactions, leads to overlapping functions of miRNAs belonging to the same families, buffering against loss of individual members (Jones-Rhoades et al., 2006). It was found that the families such as miR2118, miR395, and miR159 were highly conserved in maize and rice (Xu et al., 2017). In a few interactions, similar miRNA sequences belonging to the same families were found to target dissimilar target mRNA sequences, such as mature miRNAs from the 3′ arm of miRNA family 166 and those from the 5′ arm of miRNA family 169. In *Arabidopsis*, miR395 has been found to regulate an ATP sulfurylase and an unrelated sulfate transporter (Allen et al., 2005), and miR159 has been reported to target both *MYB101* and *MYB120* and two unrelated genes,*OPT1* and *ACS8* (Schwab et al., 2005). In maize, miR395 showed differential expression in response to salt stress, and was found to regulate *ATP sulfurylase, L-Isoaspartyl methyltransferase*, and *Beta-D-xylosidase* genes (Ding et al., 2009).

The promoter analysis of differentially expressed miRNAs indicated that they were predominantly involved in ABA signaling, the auxin response pathway, light-responsive pathways and endosperm expression. Among the 13 drought-related miRNA families, some families had common targets, i.e., zma-miR160, zma-miR390, and zma-miR393 were mostly related to the ARF (auxin response factor) transcription factor, which plays an active role in ABA and auxin mediated signaling under drought conditions (Ding et al., 2013). Similarly, miR164 targets the *NAC* transcription factor and *CUC* (cup shaped cotyledon) genes in *Arabidopsis* (Rhoades et al., 2002); these genes are responsible for root and shoot development. Furthermore, zma-miR164 showed higher expression in crown roots but not in seminal roots of maize, suggesting that it could play a crucial role in development of crown roots (Kong et al., 2014).

**Figure 7:**
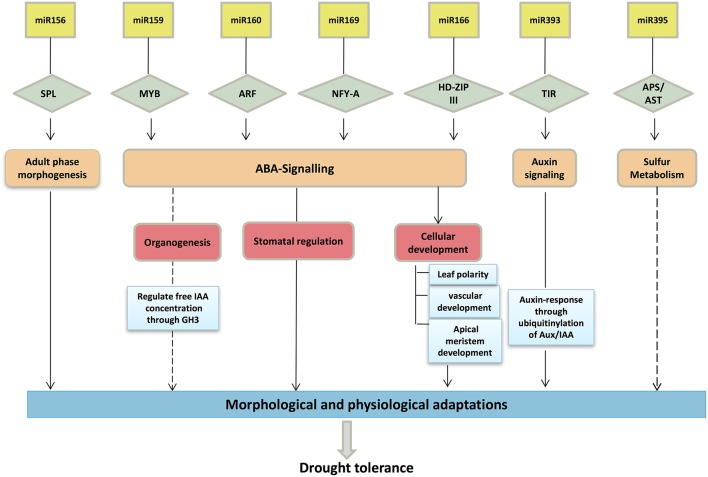
Schematic representation of important drought-related miRNAs and their targets involved in drought tolerance. The diagram depicts the possible role of targets and pathways in assigning in drought tolerance.

Members of the miR166 family generally participate in regulation of their target, *HD-Zip III* (homeodomain-leucine zipper III), which is engaged in lateral root development, initiation of axillary leaf meristems and leaf polarity (Boualem et al., 2008) (Figure 7). In maize, it was reported that the leaf polarity was controlled by a subset of miR166 family members (Nogueira et al., 2009). The regulatory mechanism of miR390 is somewhat different, as it prompts the production of tasiRNA (TAS-3 derived small interfering RNA), which targets the *ARFs* (*ARF2, ARF3*, and *ARF4*) that function in lateral root emergence and organ polarity (Nogueira et al., 2009; Meng et al., 2010). miR399 is involved in regulation of a phosphate transporter that regulates the uptake of phosphate and its translocation (Pant et al., 2008). Our *in-silico* analysis identified various target mRNAs, including *SPL* (SQUAMOSA promoter-binding-like proteins), *GAMYB* (a gibberellin- and abscisic acid-related *MYB*), *ARF* (auxin response factor), *AST* (a sulfate transporter), and *GRFs* (growth regulating factors), among others. The majority of the identified targets are also conserved across other plant species, including model systems such as *Arabidopsis* (Adai et al., 2005) and rice (Wang et al., 2004; Luo et al., 2006).

The interactions of each miRNA with a specific target are of prime importance, as these interactions can help to discern the variation in drought-induced gene expression. For example, the miR156 family regulates the expression of *SPL*, leading to plant developmental phase transitions (Wang et al., 2009; Chen et al., 2010) (Figure 6). Altered expression of this family during drought is evidence of its functional novelty in drought stress. Li et al. (2013) reported the up-regulation of miR156 at the early stage of drought stress in the maize seedlings. The regulation of SPL TFs through miR156 was also reported during somatic embryogenesis of maize (Chávez-Hernández et al., 2015). Additionally, Liu et al. (2014) reported that miR156 controlled several SPL genes during the juvenile-to-adult phase transition in maize. Over-expression of miR156 encoding the maize *Cg1* gene showed to prevent the flowering, and improved the digestibility and starch content in switchgrass (Chuck et al., 2011). Similarly, the expression of *GRF*, which plays important role in leaf growth by transforming cell proliferation, is regulated by miR396c (Kim et al., 2003).

It was interesting to note that the expression patterns of some miRNAs were genotype-specific under drought stress. In this experiment, the Type IV (opposite) pattern identified between miRNA and target mRNAs suggested negative regulation under drought stress. The up-regulation of miR396 in HKI-1532 suppressed the expression of its target, *GRF1*, whereas the up-regulation of miR396 in V-372 led to neutral expression of its target. This suggests that suppressed *GRF* expression under drought can provide tolerance by precluding leaf growth and function under stress (Figure 7). Our hypothesis is supported by the fact that *GRF8* reduces stomatal density in *Arabidopsis* (Liu et al., 2009). However, expression of miR396c and miR396d, targeting *GRFs*, was the same in both genotypes. It may be deduced that the small up-regulation of miRNAs in HKI-1532 contributes to drought tolerance. Similar results were obtained for miR396 in *Arabidopsis* (Liu et al., 2008) and tobacco (Frazier et al., 2011).

In the same way, Type I (up-regulated) miRNA-mRNA interaction was observed for miR169l, which targets transcription factor *NF-YA3* (nuclear transcription factor subunit A-3) in HKI1532. However, the same miRNA exhibited a Type IV interaction in V-372. Our hypothesis is supported by findings in *Arabidopsis*, where the role of transcription factor *NF-YA* in drought tolerance is well documented (Liu et al., 2008). Similarly, Type IV interaction was also observed for the miR159 family, targeting the transcription factor *GAMYB* that is involved in flowering development and flowering time. Furthermore, overexpression of the miR159 family during stress aids in germination under stress conditions. In *Arabidopsis*, overexpression of *MYB* transcription factors (*MYB33* and *MYB101*) provides tolerance to drought by adapting ABA hypersensitivity (Reyes and Chua, 2007). Regulation of *MYB* transcription factors by the member of miR159 was also reported in temperate maize (Li et al., 2013) and was found to play a crucial role in stress tolerance (Wang Y. G. et al., 2014).

The miR393 family that targets the *TIR1* (transport inhibitor response 1) enzyme was up-regulated in V-372. This enzyme directly participates in the ubiquitinylation of inhibitors of the auxin response pathway (Dharmasiri and Estelle, 2002). miR160, representing a Type IV interaction in HKI-1532, plays a role in ABA-auxin interaction during drought (Liu et al., 2007). Similarly, the Type IV interaction between up-regulated miR395 up-regulated and its down-regulated targets *ATP sulfurylase* and *HD-Zip* transcription factor (Buchner et al., 2004) were involved in seedling growth and seed germination in HKI-1532. This negative link indicates the role of miR395 in conferring drought tolerance. Our results are supported by the findings of studies conducted in *Arabidopsis* under drought stress, where overexpression of miR395 decreased seed growth and germination upon drought exposure (Kim et al., 2010). Similar to observations for miR156 family, Type II (down-regulated) interactions were found for *SPL* genes in both genotypes. *SPL* genes have been found to be involved in deferred blooming and the adult phase transition in *Arabidopsis* (Park et al., 2010). This can be correlated with the similar studies conducted in rice and maize (Wei et al., 2009; Zhou et al., 2010).

## Conclusions

Drought-specific miRNAs and their target mRNAs were identified from existing drought-associated microarray expression profiling data, and their expression was assayed in two contrasting subtropical maize genotypes, HKI-1532 and V-372. The promoters of all drought-related miRNAs were analyzed and confirmed the presence of important drought-responsive CREs. Eleven miRNAs belonging to nine families in HKI-1532 and seven miRNAs from three families in V-372 were differentially regulated in both genotypes. Among different miRNA-mRNA interactions, suppression of biologically important genes by miRNAs in V-372 suggests their weak performance under drought stress. It was noticed that the fact that some crucial genes being unaffected by regulatory miRNAs in tolerant genotype HKI1532 could have led to drought tolerance. Our experiment provided a clear understanding of miRNA regulation in drought response. To our knowledge, this is the first report on the role of miRNAs in regulation of drought tolerance in subtropical maize inbreds. Many of the identified candidate miRNAs and mRNAs from the present investigation could be used as potential candidates for development of drought-tolerant maize hybrids for the subtropical production system.

## Author contributions

JA and TN: conceived and designed the experiments; JA, SK, AK, BP, and MGM: performed the experiments; SR, MS, and ARR: analyzed the data; All authors contributed to manuscript preparation. All authors have read and approved the final manuscript.

### Conflict of interest statement

The authors declare that the research was conducted in the absence of any commercial or financial relationships that could be construed as a potential conflict of interest.
